# Review of the Diagnosis and Management of Pulmonary Hypertension Associated with Interstitial Lung Disease (ILD-PH)

**DOI:** 10.3390/jcm14062029

**Published:** 2025-03-17

**Authors:** Zein Kattih, Ho Cheol Kim, Shambhu Aryal, Steven D. Nathan

**Affiliations:** 1 Advanced Lung Disease and Transplant Program, Inova Heart and Vascular Institute, Inova Fairfax Hospital, Falls Church, VA 22042, USA; zein.kattih@inova.org (Z.K.); shambhu.aryal@inova.org (S.A.); 2 Department of Pulmonary and Critical Care Medicine, Asan Medical Center, University of Ulsan College of Medicine, Seoul 05505, Republic of Korea; hocheol.kim@inova.org

**Keywords:** ILD-PH, PH-ILD, interstitial lung disease, pulmonary hypertension, advanced lung disease

## Abstract

Pulmonary hypertension associated with interstitial lung disease (ILD-PH) frequently complicates the course of patients with fibrotic ILD. In this narrative review, the authors assess current diagnostic tools and management considerations in ILD-PH patients. ILD-PH is associated with increased morbidity and mortality and may be suggested by the presence of symptoms out of proportion to the extent of the ILD. There are other clues to the presence of PH in the context of ILD including the need for supplemental oxygen, a reduced DLCO especially if accompanied by a disproportionately higher forced vital capacity, imaging demonstrating an enlarged pulmonary artery or a dilated right ventricle, or objective evidence of a reduced exercise capacity. While echocardiography is one screening tool, right heart catheterization remains the gold standard for the diagnosis of PH. When appropriate, treatment with inhaled treprostinil, or possibly other pulmonary vasodilators, may be indicated.

## 1. Introduction

Interstitial lung disease (ILD) encompasses a broad group of diseases affecting the pulmonary interstitium that are characterized by varying amounts of inflammation and/or fibrosis. When accompanied by fibrosis, any of these ILDs may result in associated pulmonary hypertension (PH) which is classified by the World Symposium for Pulmonary Hypertension (WSPH) under the World Health Organization (WHO) group 3 category [1,2,3]. Patients with group 1 pulmonary arterial hypertension (PAH) may have evidence of some mild ILD, and how much ILD is needed to be regarded as group 3 PH is controversial and should be determined on a case-by-case basis. Categorization as group 1 PAH or group 3 ILD-PH is important to discern, as this may have implications for prognosis, management, and especially treatment [4]. In this narrative review, we present definitions of PH as they pertain to patients with ILD-PH, explore the pathogenesis of PH in ILD, evaluate diagnostic findings that may be suggestive of ILD-PH, and examine pharmacologic therapies and management strategies for ILD-PH patients. A focused literature search was performed to identify initial key references by utilizing the PubMed database with the following keywords: “interstitial lung disease-associated pulmonary hypertension”, “interstitial lung disease-associated AND pulmonary hypertension”, and “pulmonary hypertension related to interstitial lung disease”. Relevant systematic reviews and reviews from 2020 until 2024 were reviewed for inclusion. References from these manuscripts were also evaluated for inclusion if they provided unique information. The literature search was augmented by the authors’ own knowledge. Where relevant, references were included for original studies and prediction model studies.

## 2. Definitions

PH is currently defined by a mean pulmonary artery pressure (mPAP) > 20 mmHg. Precapillary PH is accompanied by a pulmonary vascular resistance (PVR) > 2 Woods units (WU) and a pulmonary capillary wedge pressure (PCWP) of ≤15 mmHg [3]. This latter definition was initially proposed by the 2022 European Society of Cardiology/European Respiratory Society consensus statement and recently endorsed by the 7th WSPH [1]. Many of the prior estimates of disease prevalence were based on the older definitions of PH, specifically a mPAP ≥ 25 mmHg. Severe PH in the context of lung disease has been defined as a PVR >5 WU [2]. As yet, there is no definition of mild or moderate PH in the context of ILD (ILD-PH), although it is noteworthy that “mild” or borderline PH has been shown to carry a prognosis similar to patients with more severe PH [5].

## 3. Epidemiology

### 3.1. Prevalence and Incidence of ILD-PH

Estimates of the incidence and prevalence of ILD-PH depend first on the incidence and prevalence of the underlying ILDs. The incidence of PH associated with the respective ILDs depends on the cohort studied, their disease severity, and how the PH was defined. The prevalence of ILDs in the United States in 2019 was estimated to be 654,841 cases (95% confidence interval 566,536–745,855) [6], while the estimated prevalence of associated PH ranges from as low as 15% to as high as 86% [4]. Most estimates of PH associated with ILD originate from the idiopathic pulmonary fibrosis (IPF) literature. This broad range not only reflects the variability in the timing of the identification of PH in ILD patients but also the discrepancy in diagnostic threshold and modalities used for the diagnosis. Indeed, many of these estimates were obtained prior to 2018, and are therefore under the umbrella of the older definition; specifically, a mPAP ≥ 25 mmHg and precapillary PH further defined by a PVR of ≥3 WU and a PCWP ≤ 15 mmHg [3]. One study of IPF patients listed for lung transplantation demonstrated that the prevalence of PH using the pre-2018 definition was 47.6% but increased to 73.6% under the new definition [7]. There is a paucity of literature pertaining to the prevalence of PH in non-IPF ILDs with available estimates shown in Table 1 [8,9,10,11,12,13,14,15,16,17,18,19]. Most patients with ILD will have mild-to-moderate PH, and there is a subgroup of patients who are found to have severe PH at the time of their initial right heart catheterization (RHC) [2].

### 3.2. PH in CTD-ILD

Patients with Connective Tissue Disease (CTD)-related ILD (CTD-ILD), such as scleroderma and rheumatoid arthritis, may have WHO Group 3 PH secondary to underlying lung disease, or they may have WHO Group 1 disease due to vascular involvement of their underlying CTD [1]. Therefore, defining the prevalence of WHO Group 3 PH in this group of patients remains challenging. In the Pulmonary Hypertension Assessment and Recognition of Outcomes in Scleroderma (PHAROS) study, 49 of 237 patients (20.6%) with scleroderma had PH [20]. In total, 15 of those 49 with PH had ILD-PH, which represents 6.3% of the total cohort [20]. These patients were primarily New York Heart Association (NYHA) functional class 2 (54%) and 3 (23%) [20]. In the DETECT study, 31% of patients with systemic sclerosis had PH with a mPAP ≥ 25 mmHg [21]. In a scleroderma-ILD cohort, 31% of patients had PH confirmed by RHC [15]. In rheumatoid arthritis-related patients, PH typically occurs in the setting of parenchymal lung involvement, though vascular involvement and chronic thromboembolic disease may also lead to PH [22,23]. One study estimated the prevalence of PH in patients with rheumatoid arthritis to be 14% using a resting PASP cutoff of >35 mmHg [24]. The incidence of PH by echocardiography in rheumatoid arthritis patients was 26.7% compared with 4.5% in controls, and there was a strong correlation between pulmonary artery pressure and the disease duration [22].

### 3.3. PH in Combined Pulmonary Fibrosis and Emphysema

Patients with combined pulmonary fibrosis and emphysema (CPFE) have a reported prevalence of PH 15 to 55% [25]. These patients tend to have relatively well-preserved forced vital capacities (FVC) due to the presence of concomitant hyperinflation from their emphysema [26]. Some studies have suggested that the severity of PH is worse among those with CPFE compared with both IPF and chronic obstructive pulmonary disease or emphysema alone [2,25]. Estimated systolic pulmonary artery pressures are higher in patients with CPFE than in those with isolated IPF [25]. The additional burden of emphysema, over and above a given extent of fibrosis, increases the risk of PH [25]. However, the likelihood of PH does not differ for matched extents of disease (combined fibrosis and emphysema) on HRCT (or when adjusted for DLCO) between patients with CPFE and those with fibrosis alone [25].

### 3.4. Outcomes in ILD-PH

ILD patients with PH have worse outcomes compared to those without PH [27,28]. Notably, patients with ILD-PH have higher one-year and three-year mortalities compared to those without PH [2,27,29]. One study suggested an estimated survival probability of 84%, 71%, and 66% at 1, 3, and 5 years, respectively, in patients with ILD without PH compared with 75%, 59%, and 47%, respectively, in patients with ILD-PH [29]. Patients with mild PH (mPAP 20–24 mmHg) appear to have prognoses that are not dissimilar to those with more severe PH [30]. Therefore, “mild” PH does not connote “mild” disease. Indeed, any PH in the context of ILD connotes “severe” disease given the accompanying dismal prognosis. A PVR > 5 Wood Units has been associated with a significantly worse survival compared to a PVR ≤ 5 Wood Units, resulting in this being the cut point for what is regarded as severe PH [2]. However, rather than one distinct PVR cut point, it is likely that the PVR impacts prognosis on a continuum rather than by distinct categories. Indeed, there is even data demonstrating that patients with mPAP < 20 mmHg (hence no PH by definition), but with PVRs ≥ 3, have a worse prognosis than those with lower PVRs [7,31]. The presence of PH is not only associated with greater mortality, but also with significant morbidity including a greater need for supplemental oxygen, decreased exercise tolerance, more frequent acute exacerbations, and greater health-care resource utilization [32].

## 4. Pathogenesis

**Figure 1:**
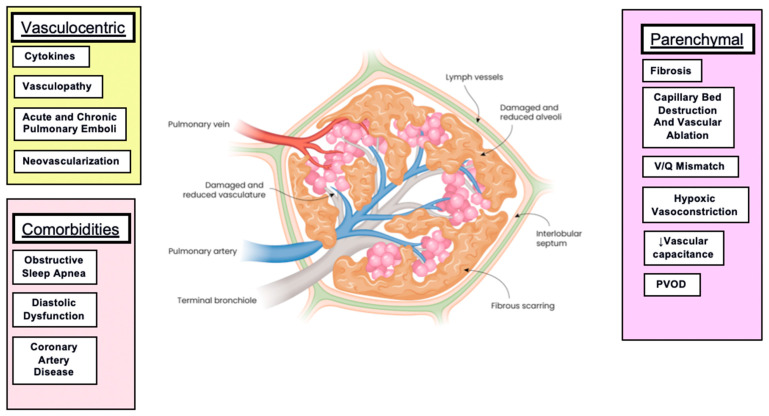
Pathogenic factors associated with the development of PH in ILD. Schematic diagram detailing the vasculocentric, parenchymal, and comorbid risk factors associated with the development of PH in ILD. ↓vascular capacitance = decreased vascular capacitance Adapted from Image © 2023 United Therapeutics Corporation. All rights reserved. Used with permission.

The mechanisms underlying the development of PH and the progression of ILD are not yet fully understood and require further investigation. Although there is an association between the extent of parenchymal involvement and pulmonary hypertension, patients with mild parenchymal involvement can still develop severe PH [33]. The pathogenesis of PH in the context of ILD is complex and multifactorial. It is not only fibrosis and vascular ablation, but other factors are involved as well [2,4,34]. Hypoxemia may cause vasoconstriction thereby contributing to the development of PH [4]. Pulmonary vascular remodeling in PH is characterized by degenerative changes and pulmonary vascular cell proliferation [35]. Pulmonary vasculopathy also features vascular pruning with loss of small pulmonary vessels [36]. The influence of comorbidities is important to recognize as these may potentially be modifiable. Specifically, heart failure, sleep-disordered breathing, and thromboembolic disease may all be contributory [2,4]. The architectural distortion that accompanies fibrosis can lead to shear stress, which can perpetuate the PH. Some of the same cytokines that are upregulated as part of the fibrotic process have also been associated with the development of PH, including endothelin, thromboxane, and transforming growth factor alpha. These may lead to vasoconstriction and vascular smooth muscle proliferation, thereby contributing to the development or worsening of PH [34,37]. There are also reports suggesting that certain genetic pathways are associated with ILD-PH [38,39]. A cartoon depiction of the factors involved in the development of PH in ILD is shown in Figure 1.

## 5. Diagnostics Suggestive of ILD-PH

**Figure 2:**
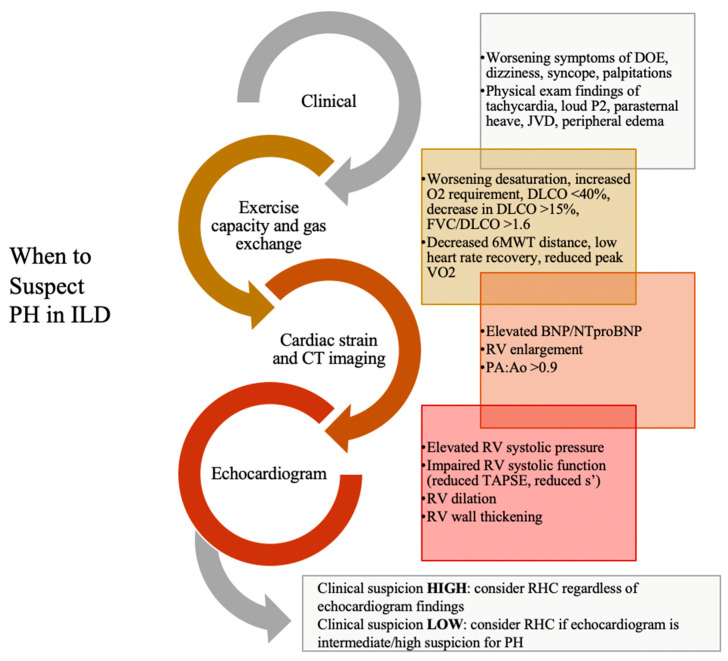
Features suggestive of PH in ILD patients. Abbreviations: 6MWT—six-minute walk test; BNP—brain natriuretic peptide; DLCO—diffusion capacity of carbon monoxide; DOE—dyspnea on exertion; FVC/DLCO—forced vital capacity/DLCO ratio; JVD—jugular venous distention; NT-proBNP—N-terminal pro b-type natriuretic peptide; PA:Ao—pulmonary artery to aorta ratio; PH—pulmonary hypertension; RHC—right heart catheterization; RV—right ventricle; TAPSE—tricuspid annular plane systolic excursion; VO2—maximum oxygen consumption.

The presence of PH in the setting of ILD may be suspected based on symptoms, physiologic testing, and abnormalities present on imaging. These findings include abnormalities on pulmonary function tests (PFT), six-minute walk test (6MWT), biomarkers, computed tomography (CT) imaging, and echocardiogram, as suggested in a multidisciplinary Delphi study of 16 pulmonologists with ILD and PH expertise [40]. The variables and tests that may raise the index of suspicion for PH complicating ILD are shown in Figure 2.

### 5.1. Clinical Features

The symptoms of ILD-PH may be difficult to distinguish from the symptoms of the underlying ILD. These may include worsening shortness of breath that appears to be out of proportion to the severity of the underlying lung disease, especially if such worsening occurs in the context of an unchanged FVC [2]. Symptoms of syncope or pre-syncope are late signs that occur more commonly in group 1 PAH. Clinical features may be subtle, such as an increased P2 heart sound. A murmur of tricuspid regurgitation may be appreciated while more overt evidence of right heart failure may be seen in the later stages of disease progression [41].

### 5.2. Biomarkers

Patients with ILD-PH may have elevated brain natriuretic peptide (BNP) or N-terminal pro b-type natriuretic peptide (NT-proBNP) levels or elevated troponin levels suggestive of right ventricular dysfunction [42]. Elevated BNP levels have been shown to predict outcomes in patients with ILD. In a retrospective review of 131 patients with IPF, BNP was an independent predictor of prognosis, and patients with elevated BNP had a shorter survival time compared to those with normal BNP levels (70.5% vs. 23.7% 1-year mortality) [43]. In a review of 90 ILD patients, BNP level ≥ 20 pmol/L was correlated with increased mortality independent of age, sex, and pulmonary function (hazard ratio 2.93), and these patients had a 14-fold increase in mortality compared with patients with BNP < 4 pmol/L [44]. How much of this elevation in BNP was from underlying PH versus concurrent heart failure was uncertain. Importantly, a normal BNP or NT-proBNP does not rule out underlying PH in ILD patients. The specific role of biomarkers in ILD-PH is not as well defined as in WHO Group 1 disease, particularly in patients with concurrent renal dysfunction, obesity, or left heart disease.

### 5.3. Physiologic Testing

Pulmonary function testing is obtained as the standard of care in patients with ILD. Variables from these can provide clues to the presence of PH. It is important to note that there is a poor correlation between the extent of the restrictive physiology and the presence and severity of any underlying PH. The single-breath diffusing capacity for carbon monoxide (DLCO) is the one PFT variable that is associated with the presence of PH. A reduced DLCO (e.g., <40%) or an elevated FVC% predicted/diffusion lung capacity for carbon monoxide percent predicted (FVC%/DLCO%) ratio might indicate underlying PH. In one cohort of systemic sclerosis patients, 22% of patients with an FVC%/DLCO% greater than 1.4 had concurrent PH compared to only 2% of patients with a ratio < 1.4 [45]. While various thresholds have been suggested in the literature, these variables should rather be viewed on a continuum. Specifically, the lower the DLCO% and the higher the FVC%/DLCO% ratio, the greater the likelihood of underlying PH.

### 5.4. Functional Testing

The 6MWT is commonly used in the standard of care management of ILD patients. There are multiple clues from variables captured during the 6MWT which can indicate underlying PH. Generally, patients with ILD-PH walk less, require more oxygen, desaturate more, and have a lower heart rate recovery than non-PH ILD patients. Heart rate recovery is the difference between the heart rate at the end of the walk and the heart rate one minute into recovery. A lower heart rate recovery has been shown to correlate with worse outcomes in IPF patients and a heart rate recovery rate of <13 has been associated with the presence of PH [46].

Although not routinely obtained, cardiopulmonary exercise testing can be a useful test in patients with ILD to identify an exhausted circulatory reserve which might be suggestive of PH. Findings such as a reduced oxygen pulse, a low maximal oxygen consumption, an unchanged or decreased partial pressure of carbon dioxide during exercise, and a preserved breathing reserve can all suggest a contribution to exercise limitation from PH [47].

### 5.5. CT Imaging

**Figure 3:**
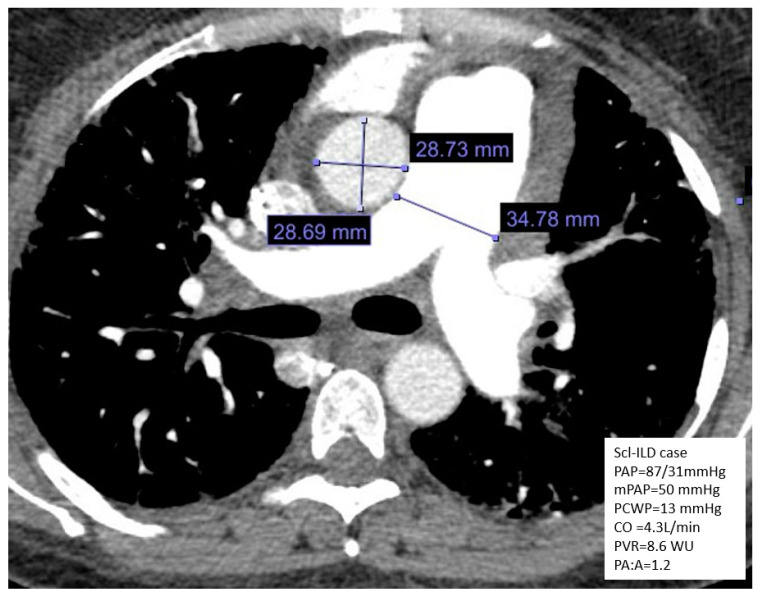
Axial view of CT angiogram of the chest with measurement of the pulmonary artery and aorta in a patient with ILD demonstrating a pulmonary artery to aorta ratio of 1.2. The patient’s hemodynamics based on right heart catheterization are shown in the bottom right square. Abbreviations: mPAP—mean pulmonary artery pressure; PAP—pulmonary artery pressure; PA:A—pulmonary artery to aorta ratio; PVR—pulmonary vascular resistance; Scl-ILD—scleroderma-related interstitial lung disease; PCWP—pulmonary capillary wedge pressure.

Imaging of the lungs via high-resolution CT is another test that is obtained in the routine diagnosis and follow-up of patients with ILD. Radiographic evidence of an enlarged pulmonary artery (PA) diameter, which is best evaluated through the PA to aorta diameter ratio (>1), or the right ventricle to left ventricle ratio on axial CT images (Figure 3) of the chest should raise suspicion for underlying PH [41].

### 5.6. Echocardiography

Echocardiographic evidence of PH may include an elevated estimated right ventricular systolic pressure, impaired right ventricular systolic function as suggested by a reduced tricuspid annual plane systolic excursion or S’, and evidence of right ventricular hypertrophy and remodeling such as with right ventricular dilation or wall thickening [48]. PH may be present despite a normal right ventricular systolic pressure on an echocardiogram, and echocardiography may be inaccurate in estimating pulmonary pressures [49]. Thus, while echocardiography is a good noninvasive screening tool for PH, results must be placed in a clinical context, and right heart catheterization may still be considered if clinical suspicion for PH remains high, despite an echocardiogram suggesting a low probability of PH [40,50].

### 5.7. Prediction Models

There have been a number of composite models proposed which incorporate elements of the modalities presented above. These have been developed to provide a more accurate prediction of PH in ILD patients. Select models are outlined in Table 2 [42,47,51,52,53,54,55,56,57,58], and a more comprehensive review of these predictive models is outlined in a narrative review by Arvanitaki and colleagues [59].

### 5.8. Hemodynamic Profile

RHC is the gold standard for the diagnosis of PH since this provides the direct measurement of the hemodynamic variables necessary to diagnose PH and pre-capillary PH [3]. Precapillary PH is defined by a mPAP > 20 mmHg, pulmonary artery wedge pressure ≤ 15 mmHg, and a PVR > 2 WU. IPF patients have an increased predisposition for comorbidities including heart failure and sleep-disordered breathing [2]. In patients suspected to have concurrent WHO group 2 disease, the use of volume challenge maneuvers or exercise during RHC may be considered [48]. Patients with ILD-PH tend to have mild to moderate PH, but any PH in the context of ILD is associated with a poor prognosis.

### 5.9. Distinguishing PAH from PH Due to Lung Disease

PH in patients with ILD can be considered on a continuum between WHO Group 1 and WHO Group 3 disease [4]. Evaluation of the extent of lung disease, hemodynamic profile, PFTs, and the presence of additional risk factors for PH can favor one category over the other [4].

Moderate-to-severe pulmonary hypertension is more suggestive of WHO group 1 disease, while more mild-to-moderate PH suggests WHO group 3 disease [4]. Patients with only mild impairment on their spirometry and a low or very low DLCO in comparison likely have a larger contribution from WHO group 1 disease, particularly when high-resolution CT of the chest demonstrates only mild or perhaps even moderate parenchymal lung disease [60]. Conversely, patients with moderate to extensive fibrosis and PFTs with moderate or severe restriction are best categorized as WHO group 3 disease [60].

## 6. Management of ILD-PH

**Figure 4:**
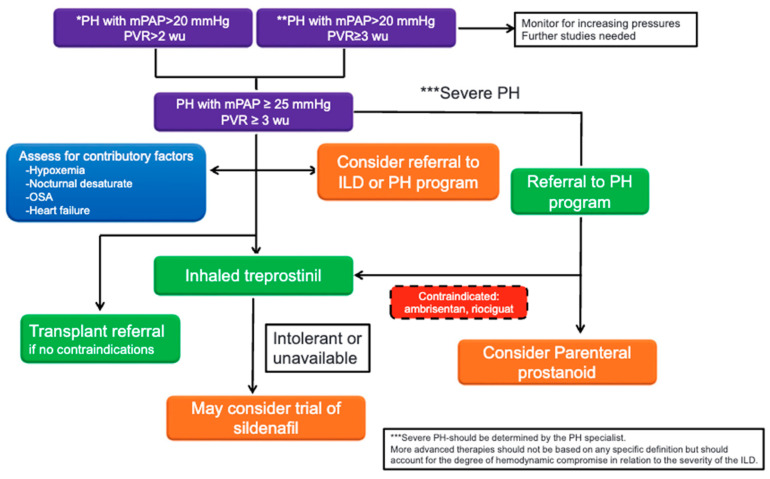
Algorithm for the management of patients with pulmonary hypertension associated with interstitial lung disease. * 2022 European Society of Cardiology and the European Respiratory Society definition of pulmonary hypertension ** 6th World Symposium on Pulmonary Hypertension Definition of Pulmonary Hypertension. *** Severe PH should be determined by the PH specialist. ILD = interstitial lung disease; mPAP = mean pulmonary artery pressure; OSA = obstructive sleep apnea; PH = pulmonary hypertension; PVR = pulmonary vascular resistance; wu = Wood units. Reprinted with permission of the American Thoracic Society. Copyright © 2024 American Thoracic Society. All rights reserved. Nathan SD. 2023. Progress in the Treatment of Pulmonary Hypertension Associated with Interstitial Lung Disease. Am J Respir Crit Care Med. 208(3):238–246. [63] The American Journal of Respiratory and Critical Care Medicine is an official journal of the American Thoracic Society.

The management of ILD-PH should always consider the management of the underlying ILD as one of the first steps. Immunosuppression may be indicated in patients with an underlying inflammatory component to their ILD (e.g., CTD-ILD). However, it is noteworthy that in ILDs which are purely inflammatory, there is very little data attesting to the prevalence and impact of PH. If the inflammatory component is reversible, then it makes intuitive sense that any accompanying PH will be reversible too. Fibrotic lung disease should be addressed with consideration for the institution of an antifibrotic therapy [61,62]. There are no data demonstrating that antifibrotic therapy will reverse or slow the development of PH. The need for supplemental oxygen, including sleep and exertion, should be assessed serially. There should also be a heightened index of suspicion and screening for any comorbid conditions which might be contributory. If present, management of sleep apnea, thromboembolic disease, or heart failure should be optimized [48]. Referral to pulmonary rehabilitation and early consideration for a lung transplantation evaluation in appropriate candidates is strongly encouraged. An algorithm for the management of patients with ILD-PH is shown in Figure 4.

## 7. Pharmacological Management of ILD-PH

### 7.1. Endothelin Receptor Antagonists (ERAs)

The ERAs are the oldest form of oral therapy approved for the treatment of PAH. There are currently three agents that are available, including bosentan, ambrisentan, and macitentan. These have all been subjected to clinical trials in IPF for their purported antifibrotic properties, but unfortunately, all three of these studies were negative [64,65,66]. Raghu and colleagues in the Randomized Placebo-Controlled Study to Evaluate Safety and Effectiveness of Ambrisentan in IPF (ARTEMIS-IPF) evaluated ambrisentan in IPF patients, 32 of whom had PH; however, the study was terminated early due to lack of efficacy in time to clinical worsening [64]. The later ARIES-3 open-label trial of ambrisentan in adult patients with PH due to Group 1, 3, 4, and 5 PH found no improvement in 6MWT distance in the subpopulation of patients with ILD, and, in fact, demonstrated a mean decline of 23 m on 6MWT [67].

The most robust of the ERA studies specifically targeting ILD-PH was the bosentan in pulmonary hypertension with fibrotic idiopathic interstitial pneumonia (BPHIT study) [66]. This study was a prospective, double-blind, controlled study of fibrotic ILD patients randomized in a 2:1 fashion to bosentan or placebo [66]. This study was negative based on the primary endpoint of reduction in the PVR index at 16 weeks. There were also 20 negative secondary endpoints with none showing even a trend to benefit [66].

### 7.2. Riociguat

Riociguat is a soluble guanylate stimulator approved for both PAH and chronic thromboembolic PH. It was shown in an open-label trial to be associated with improved cardiac output, PVR, and 6MWT distance at 12 weeks [68]. This was the basis for a subsequent phase 3 study (the Safety of Riociguat in Patients with Symptomatic Pulmonary Hypertension Associated with Idiopathic Interstitial Pneumonias- RISE-IIP), which was a double-blind, randomized, placebo-controlled study of 147 patients with RHC confirmed PH and an FVC of at least 45% of predicted [69]. Unfortunately, the study was terminated early due to increased adverse events in the riociguat group and increased mortality in patients treated with riociguat [69]. As a result of this trial, riociguat is contraindicated in patients with ILD-PH.

### 7.3. Phosphodiesterase 5 (PDE5) Inhibitors

There have been a number of retrospective and open-label trials that have evaluated the role of sildenafil [70,71,72,73,74], and at least one of tadalafil [71,75] in ILD-PH patients. Evidence from these retrospective studies has suggested the potential for improvement in the 6MWT [70,73,74], BNP [44], ventilation–perfusion matching, and oxygenation [72] with sildenafil. However, two prospective randomized controlled studies of sildenafil did not show a benefit in the 6MWT distance and Borg score [76,77]. Notably, sildenafil did improve oxygen saturation and quality of life as secondary endpoints in this study [77]. There have also been a number of studies of sildenafil in IPF populations enriched for the presence of PH by the inclusion criterion of a DLCO < 35% of predicted. The Sildenafil Trial of Exercise Performance in IPF (STEP-IPF) study, while a negative study based on the primary endpoint of a 20% improvement in the 6MWT distance, did have a number of secondary endpoints that were positive, including arterial oxygenation, DLCO, degree of dyspnea, and quality of life in favor of sildenafil [77]. In addition, a recent study reported that the use of PDE5 inhibitors was associated with favorable outcomes in severe PH associated with ILD [78].

### 7.4. Prostanoids

The prostanoids were the first class of agents approved for the treatment of PAH. In 1999, inhaled and intravenous prostanoids together with inhaled nitric oxide were studied in ILD patients and were shown to be associated with an improvement in gas exchange [79]. In 2007, Krowka and colleagues evaluated inhaled iloprost in 51 patients with IPF and demonstrated no difference in 6MWT, NYHA functional class, dyspnea score, or oxygen saturation with the drug [80]. Both epoprostinil [81] and treprostinil [82] have been evaluated in patients with ILD-PH. Saggar and associates reported improvements in multiple echocardiographic domains, 6MWT, and patient-reported outcomes in a prospective open-label study of parenteral treprostinil in patients with severe ILD-PH (mPAP ≥ 35 mmHg) [82]. One case series described the use of intravenous prostacyclins followed by maintenance inhaled prostacyclins for three patients with ILD-PH presenting as RV failure [38]. However, the most robust data pertaining to prostanoid therapy resulted from the INCREASE study of inhaled Treprostinil [83].

The INCREASE trial was a double-blind, randomized, multi-center, placebo-controlled trial of inhaled Treprostinil in patients with ILD-PH, including those with CPFE [83]. The trial enrolled 326 patients and demonstrated a 31.12 m placebo-corrected improvement in the 6MWT distance at 16 weeks with the use of inhaled Treprostinil [83]. There was also a 15% reduction in NT-proBNP levels in the treatment arm compared to a 46% increase in the placebo group, while clinical worsening occurred in 22.7% of the active treatment group vs. 33.1% of placebo patients (*p* = 0.04) [83]. Based on these results, inhaled Treprostinil became the first FDA-approved drug specifically for the treatment of ILD-PH.

There have been a number of post hoc analyses of the INCREASE trial, which have further explored the benefits of inhaled Treprostinil [84,85,86]. Less clinical worsening events with inhaled Treprostinil have been further validated in two of these analyses that have evaluated multiple progression events and employed a win ratio approach [85,86]. A potential direct antifibrotic effect of inhaled Treprostinil also emerged from the INCREASE trial [85]. Spirometry was performed in all participants as a safety endpoint, but what was noted was an apparent benefit in FVC with inhaled Trepostinil. Specifically, there was a placebo-corrected difference in the FVC of 28.5 mL at week 8 and 44.4 mL at week 16 favoring inhaled Treprostinil. This finding was most evident in patients with idiopathic interstitial pneumonia, especially IPF [85]. Another post hoc analysis of the INCREASE trial also suggested a long-term survival benefit of inhaled Treprostinil using models of survival that are commonly employed in the oncology literature [87].

A subsequent multicenter, non-randomized, open-label trial of ILD-PH patients was conducted in a Japanese population to evaluate change in pulmonary vascular resistance (PVR) index and peak 6MWT distance [88]. This trial of 20 patients demonstrated a PVR index decrease of 40.1% and a peak 6MWT distance increase of 13 m, reinforcing the efficacy shown in the INCREASE trial [88]. Interestingly, there also appeared to be a small improvement in the FVC in this population as well as a decrease in Krebs von den Lungen-6 levels, a fibrotic biomarker [88].

Based on these results, inhaled Treprostinil has emerged as the first-line treatment for ILD-PH. Inhaled Treprostinil allows for direct delivery of medications to the lungs, reducing the risk of systemic symptoms such as systemic vasodilation. Side effects that may limit the use of inhaled Treprostinil include cough, and modification of route of administration (via nebulizer or dry powder inhalation) may mitigate some side effects. PDE5 inhibitors such as sildenafil and tadalafil are also utilized, as are prostanoids on a case-by-case basis. ERAs and riociguat are generally contraindicated in these patients. As further evidence emerges, the management of these patients will continue to evolve. A suggested treatment approach is shown in Figure 4 [63].

### 7.5. Clinical Trials in ILD-PH

**Figure 5:**
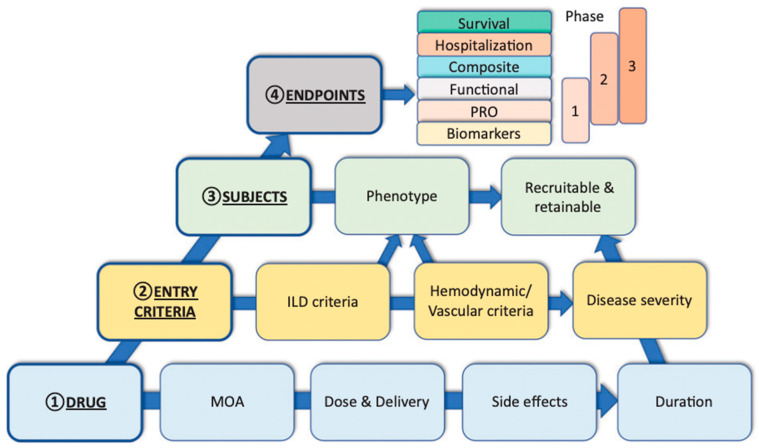
Conceptual framework for clinical trials in ILD-PH. Factors to consider in the design of future clinical trials for pulmonary hypertension associated with interstitial lung disease (ILD-PH). The diagram depicts four different pillars and how their respective variables interrelate and may be considered to optimize ILD-PH trial feasibility. MOA = mechanism of action; PRO = patient-reported outcome. Reprinted with permission of the American Thoracic Society. Copyright © 2024 American Thoracic Society. All rights reserved. Nathan SD. 2023. Progress in the Treatment of Pulmonary Hypertension Associated with Interstitial Lung Disease. Am J Respir Crit Care Med. 208(3):238–246. [63] The American Journal of Respiratory and Critical Care Medicine is an official journal of the American Thoracic Society.

The prevalence of ILD and the risk of PH developing in many of these patients amplifies the need for further therapies to address this largely unmet need. There are many medications approved for PAH and only one therapy approved for ILD-PH, despite its much greater prevalence and more dire prognosis. There are numerous considerations in the design of ILD-PH trials including but not limited to the patient phenotype most likely to respond, the optimal duration of the study, and the best endpoint(s) to employ. A conceptual framework of these is outlined in Figure 5 [2,89].

Although there are well-established patient-reported outcome (PRO) questionnaires for both PAH and IPF, there are as yet no PROs specific to ILD-PH; hence this is another area for future research. Finally, further clinical data are required in patients meeting the updated definition of PH to determine the role of treatment in this subgroup. Trials are ongoing to evaluate the safety and efficacy of new therapies (Table 3).

## 8. Future Directions

**Figure 6:**
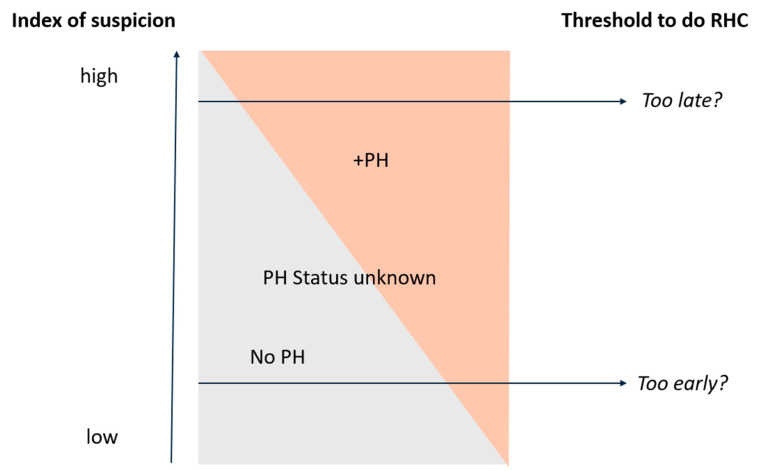
Conceptual depiction of the threshold to perform an RHC and the likelihood of demonstrating its presence. The “Sweet spot” to recommend an RHC remains uncertain.

There is a large unmet need for the treatment of ILD-PH and a significant knowledge gap in how best to phenotype these patients [50]. How best to integrate and evaluate the extent of the parenchymal lung disease, and the hemodynamics is also uncertain. Whereas there has been reliance on lung function testing to evaluate the former, there is increasing evidence that lung imaging has a critical role. In this regard, functional respiratory imaging has promise as an avenue to explore the extent of the fibrosis as well as details of the associated vascular involvement. The optimal screening strategies with further development and validation of risk assessment tools for PH in ILD patients require further study. This is the subject of an ongoing prospective study (PHinder study NCT05776225). At what point in a patient’s clinical course should an RHC be obtained also remains uncertain. The level of suspicion juxtaposed to the likelihood of demonstrating PH is depicted in a concept figure (Figure 6). In addition, if ILD patients do not have PH on RHC, when is the optimal time to repeat their RHC, if ever? While the thresholds defining PH have been lowered, there is no data on what to do with patients who previously would not have been categorized as having PH (i.e., those with PVR 2–3 WU). In addition, controversy remains as to whether to treat ILD-PH patients with PVR of 3–4 WU. While most of the epidemiologic and prognostic data pertaining to ILD-PH is derived from IPF patients, further data on the prevalence and progression of disease within the other ILD subtypes is currently lacking. In summary, there are abundant opportunities and a need for further research into many aspects of ILD-PH [2].

## 9. Conclusions

PH in the presence of ILD is associated with significant morbidity and mortality, and early recognition is crucial for the management of these patients. Clinical, biomarker, and physiologic data, may suggest the presence of PH in ILD patients while echocardiography provides further supportive evidence, but right heart catheterization remains the gold standard for the diagnosis of precapillary PH. There is now an available treatment option for ILD-PH which is currently only approved in a few countries, namely, inhaled Treprostinil. Other off-label therapies such as intravenous and/or subcutaneous prostanoids may be considered on a case-by-case basis, but these should only be implemented at expert centers. Further research is needed in phenotyping patients with ILD-PH and in evaluating screening tools and novel treatment modalities.

## Figures and Tables

**Table 1:** Estimated prevalence of pulmonary hypertension according to interstitial lung disease subtype.

Subtype	Reference	PH Out of Total Cohort	Prevalence	Location	Diagnostic Modality of ILD	Diagnostic Modality of PH
Pooled Prevalence of ILD	Ang et al., 2024 (pre-6th WSPH definition) [8]		36%	International	Variable	RHC
	Ang et al., 2024 (6th WSPH definition) [8]		40%	International	Variable	RHC
Idiopathic pulmonary fibrosis	Shorr et al., 2007 [10]	1163/2525	46%	UNOS Database	Variable	RHC
	Nathan et al., 2008 [11]	17/44	39%	United States	HRCT +/− pathology	RHC
	Todd et al., 2010 [12]	12/41	29%	United States	Clinical + CT +/− pathology	RHC
	Raghu et al., 2015 [13]	68/488	14%	Multicenter	Either surgical lung biopsy showing UIP or HRCT with definite UIP	RHC
	Tyagi et al., 2021 [14]	18/38	47%	India	Clinical + HRCT +/− pathology	TTE
Systemic sclerosis-ILD (SSc-ILD)	Young et al., 2019 [15]	29/93	31%	United States	ACR/EULAR classification criteria for SSc + HRCT showing ILD	RHC
CTD-ILD other than systemic sclerosis	Todd et al., 2010 [12]	9/14	64%	United States	Clinical + CT +/− pathology	RHC
	Cottin et al., 2011 [16]	28/61	47%	France	HRCT + PFT +/− clinical +/− exclusion of ILD with known etiology and exclusion of CTD	TTE
	Tyagi et al., 2021 (Autoimmune ILD) [14]	33/75	44%	India	Clinical + HRCT +/− pathology	TTE
Combined pulmonary fibrosis and emphysema	Cottin et al., 2005 [18]	61	47%	France	Emphysema on HRCT + diffuse parenchymal lung disease on HRCT	TTE
Nonspecific interstitial pneumonia (NSIP)	King et al., 2018 [17]	11/35	31%	United States	Biopsy-proven NSIP	RHC
Chronic hypersensitivity pneumonitis	Oliveira et al., 2014 [19]	22/50	44%	Brazil	Clinical + HRCT +/− pathology	RHC
	Tyagi et al., 2021 [14]	40/77	52%	India	Clinical + HRCT +/− pathology	TTE

Abbreviations: ACR/EULAR—American College of Rheumatology/European League of Rheumatology; CT—computed tomography of the chest; CTD-ILD—connective tissue disease-related ILD; HRCT—high resolution computed tomography of the chest; ILD—interstitial lung disease; PFT—pulmonary function test; pre-6th WSPH definition—pre-6th World Symposium on Pulmonary Hypertension Definition of PH; PH—pulmonary hypertension; RHC—right heart catheterization; TTE—transthoracic echocardiogram; UIP—usual interstitial pneumonia; UNOS—United Network for Organ Sharing.

**Table 2:** Select prediction models for PH in ILD patients.

Reference	Population	Parameter	Prediction Equation	Outcome	*p*-Value (If Available)
Zisman et al., 2007 [51]	IPF	SpO2, FVC, DLCO	−11.9 + 0.272 × SpO2 + 0.0659 × (100−SpO2) 2 + 3.06 × (% FVC/% DLco)	mPAP	PPV 71% NPV 81%
Furukawa et al., 2018 [52]	IPF	DLCO, PA/Ao ratio on CT chest, PaO2	Simple scoring system assigning 1 point to each variable (DLCO < 50%, PA/Ao > 0.9, PaO2 < 80 Torr)	mPAP	C-index: 0.772, *p* < 0.001 OR of variables: DLCO 3.6, *p* < 0.001 PA/Ao 2.6, *p* = 0.012 PaO2 2.2, *p* = 0.030
Bax et al., 2018 [53]	ILD	PASP, right atrial area, early diastolic pulmonary regurgitant velocity, FAC, RV:LV ratio, eccentricity index	Stepwise echocardiographic algorithm	mPAP ≥ 35 mmHg	Score ≥ 7 predicting severe PH Sensitivity 89% Specificity 71% PPV 68% NPV 90%
Sonti et al., 2019 [54]	IPF	sPAP, FVC/DLco, PA/A ratio	mPAP = −14 + 20.3 × (PA:A ratio) + 2.6 × (FVC/DLCO) + 0.3 × (RVSP)	mPAP	Sensitivity 80% Specificity 68.6% PPV 56% NPV 87.2%
Sobiecka et al., 2020 [55]	ILD	Age, TLC/DLCO, 6MWD, saturation at 6 min	Scoring system including age > 53 years, TLC/DLCO > 1.67, 6MWD < 507.5 m, saturation at 6 min < 93% (max score 10)	sPAP by echocardiography	AUC 0.867 Cutoff of 6 points yields: Sensitivity 66% Specificity 94% PPV 90% NPV 78%
Refini et al., 2021 [56]	IPF	sPAP, PA area by HRCT, and ratio of segmental artery to adjacent bronchus of L1/2	Composite index of these variables	mPAP	R^2^ = 0.53, *p* = 0.0009 Sensitivity 100% Specificity 53% PPV 71% NPV 100%
Parikh et al., 2023 [58]	ILD	History, physical exam, 6MWD, DLCO, PA/A ratio, NT-proBNP	ILD-PH Detection tool (DLCO < 40%, 6MWD < 350 m, NTprBNP > 300 pg/mL, PA enlargement, physical exam for PH, supplemental O2, syncope/presyncope, CTD or sarcoid)	PH	Score of ≥7 predicting PH Sensitivity 87% Specificity 86%
Joseph et al., 2023 [47]	ILD	Gas exchange-derived pulmonary vascular capacitance (GXcap), RVSP, FVC/DLCO	GXcap < 416 mL*mmHg, FVC/DLCO > 1.7, RVSP > 73	ILD-PH	AUC 0.94 Sensitivity 86% Specificity 93%
Nathan et al., 2024 [57]	IPF	FVC/DLCO, oxygen saturation nadir, race, 6MWD	FORD calculator (continuous) and FORD index (simple point-score system)	PH (pre-6 WSPH definition)	Derivation cohort AUC 0.75 Validation cohort AUC 0.69

Abbreviations: 6MWD—six-minute walk test distance; AUC—area under the curve; CTD—connective tissue disease; DLCO—single breath diffusing capacity for carbon monoxide; FAC—fractional area change; FVC—forced vital capacity; ILD—interstitial lung disease; IPF—idiopathic pulmonary fibrosis; HRCT—high resolution computed tomography; mPAP—mean pulmonary artery pressure; NTproBNP—N-terminal pro b-type natriuretic peptide; NPV—negative predictive value; OR—odds ratio; PA/A ratio or PA/Ao ratio—pulmonary artery to aorta ratio; PaO2—partial pressure of oxygen; PASP—pulmonary artery systolic pressure; pre-6th WSPH definition—pre-6th World Symposium on Pulmonary Hypertension Definition of PH; PH—pulmonary hypertension; PPV—positive predictive value; RV:LV ratio—right ventricle to left ventricle ratio; RVSP—right ventricular systolic pressure; SPAP—systolic pulmonary artery pressure; SpO2—oxygen saturation; TLC—total lung capacity.

**Table 3:** Ongoing pharmacologic clinical trials in ILD-PH.

Clinicaltrials.gov Number	Drug	Administration	Mechanism of Action	Phase of Study	Design	Treatment Period (Weeks)	Primary End Point
NCT05176951	Treprostinil palmitil	80 μg daily DPI titrated up to 640 μg	Inhaled prostanoid	2	RCT, double-blind, multicenter, placebo-controlled	16	Safety/tolerability; Change from baseline in 6MWT SpO2
NCT06129240	LIQ861 (treprostinil)	Variable QID DPI dosing	Inhaled prostanoid	3	Open-label ASCENT extension, multicenter	52	Safety/tolerability
NCT04691154	L606 (liposomal treprostini)	Twice daily aerosolized	Aerosolized liposomal prostanoid	3	Open-label extension	48	Safety/tolerability
NCT05128929	H01 (Hymecromone)	80 μg daily DPI titrated up to 640 μg	Inhibitor of hyaluronan synthesis	2	SATURN- RCT, double-blind, placebo-controlled	24	Change in PVR by RHC; safety/tolerability
NCT02036970	Bardoxolone methyl	Variable QID DPI dosing	Inducer of Nrf2 and suppressor of NF-kB	2	RCT, double-blind, placebo-controlled	16	Mean change in 6MWD
NCT06475781	Murivadelgat	Twice daily aerosolized	Aldehyde dehydrogenase 2 activator	2	WINDWARD- RCT, double, blind, multinational, placebo-controlled	12	Mean change in PVR by RHC at week 12
NCT06635850	Mosliciguat	80 μg daily DPI titrated up to 640 μg	Soluble guanylate cyclase (sGC) activator	2	PHocus- RCT, double-blind, multicenter, placebo-controlled	24	Change in PVR by RHC at 26 weeks

## Abbreviations

6MWT—six-minute walk test; BNP—brain natriuretic peptide; CPET—cardiopulmonary exercise test; CT—computed tomography; DLCO—diffusion capacity for carbon monoxide; ERA—endothelin receptor antagonists; FEV1—forced expiratory volume in one second; FVC—forced vital capacity; ILD—interstitial lung disease; ILD-PH—pulmonary hypertension associated with ILD; IPF—idiopathic pulmonary fibrosis; mPAP—mean pulmonary arterial pressure; NT-proBNP—N-terminal pro b-type natriuretic peptide; NYHA—New York Heart Association; PA—pulmonary artery; PAH—pulmonary arterial hypertension; PDE5—Phosphodiesterase 5; PFT—pulmonary function test; PH—pulmonary hypertension; PRO—patient-reported outcomes; PVR—pulmonary vascular resistance; RHC—right heart catheterization; WHO—World Health Organization; WU—Wood Units
